# Gut microbiota dynamics and fecal SCFAs after colonoscopy: accelerating microbiome stabilization by *Clostridium butyricum*

**DOI:** 10.1186/s12967-024-05031-y

**Published:** 2024-03-01

**Authors:** Zhenhui Chen, Lu Yu, Jiaxin Liu, Jingjing Kong, Xiaoshi Deng, Xiaotong Guo, Jiamin Shan, Daixuan Zhou, Wendan Li, Yangfan Lin, Wanwen Huang, Weisen Zeng, Xinlong Shi, Yang Bai, Hongying Fan

**Affiliations:** 1 Department of Microbiology, Guangdong Provincial Key Laboratory of Tropical Disease Research, School of Public Health, Southern Medical University, Guangzhou, China; 2 Department of Radiation Oncology, Nanfang Hospital, Southern Medical University, Guangzhou, China; 3 Guangdong Provincial Key Laboratory of Gastroenterology, Inst. Of Gastroenterology of Guangdong Province, Department of Gastroenterology, Nanfang Hospital, Southern Medical University, Guangzhou, China; 4 Department of Cell Biology, School of Basic Medicine, Southern Medical University, Guangzhou, China; 5 Department of Colorectal Surgery, Gansu Provincial Hospital, Lanzhou, China

**Keywords:** Gut microbiome, Colonoscopy, *Clostridium butyricum*, Short-chain fatty acids, Probiotics

## Abstract

**Background:**

Colonoscopy is a classic diagnostic method with possible complications including abdominal pain and diarrhoea. In this study, gut microbiota dynamics and related metabolic products during and after colonoscopy were explored to accelerate gut microbiome balance through probiotics.

**Methods:**

The gut microbiota and fecal short-chain fatty acids (SCFAs) were analyzed in four healthy subjects before and after colonoscopy, along with seven individuals supplemented with *Clostridium butyricum*. We employed 16S rRNA sequencing and GC–MS to investigate these changes. We also conducted bioinformatic analysis to explore the *buk* gene, encoding butyrate kinase, across *C. butyricum* strains from the human gut.

**Results:**

The gut microbiota and fecal short-chain fatty acids (SCFAs) of four healthy subjects were recovered on the 7th day after colonoscopy. We found that *Clostridium* and other bacteria might have efficient butyric acid production through bioinformatic analysis of the *buk* and assessment of the transcriptional level of the *buk*. Supplementation of seven healthy subjects with *Clostridium butyricum* after colonoscopy resulted in a quicker recovery and stabilization of gut microbiota and fecal SCFAs on the third day.

**Conclusion:**

We suggest that supplementation of *Clostridium butyricum* after colonoscopy should be considered in future routine clinical practice.

**Supplementary Information:**

The online version contains supplementary material available at 10.1186/s12967-024-05031-y.

## Introduction

Colonoscopy is a physical and diagnostic method for colonic disease or routine physical examinations [1, 2]. There are 100 million colonoscopies performed in China per year. Bowel preparation is performed half a day before colonoscopy and consists of large doses of laxatives to evacuate most of the stool from the colon and obtain a clear picture during screening [3, 4]. Patients experience the excretion of watery feces 10–20 times [3]. During colonoscopy, a large amount of physiological saline is used to thoroughly flush the intestinal tract [2]. These processes cause drastic changes in gut microbiota and nearly one-third of adults and 10% of children experience complications during and after colonoscopy including abdominal pain and diarrhoea [5–7]. Therefore, it is necessary to explore the changes in the gut during and after colonoscopy and devise a strategy to reduce adverse reactions.

Anaerobic microorganisms in the mammalian large intestine ferment and produce a large number of short-chain fatty acids (SCFAs) including acetic acid, propionic acid, and butyric acid [8]. These SCFAs provide energy to the colon with butyric acid being the main energy source for intestinal mucosal cells [9, 10]. Butyric acid also promotes intestinal mucosal repair and functional recovery in human intestinal cells [11–13]. *Clostridium*, *Eubacterium*, *Fusobacterium*, and *Bacteroidaceae* are anaerobic bacteria which efficiently produce butyric acid [14–16]. The final steps in butyrate synthesis occurs through two main pathways: the butyryl-CoA:acetate CoA-transferase (*but*) pathway and the butyrate kinase (*buk*) pathway [17, 18]. Briefly, butyryl-CoA:acetate CoA-transferase transfers the CoA moiety to external acetate forming acetyl-CoA and butyrate, while butyrate kinase converts butyryl-CoA into butyrate [19]. Bacteria contain two *buk* homologs: *buk I* and *buk II* [20]. Previous detection of *but* and *buk* in gut microbiota has mainly focused on one type of bacteria or a genus of bacteria [21–23]. A systematic review of cultivated gut bacteria with the *buk* gene is needed given the important role of the gut microbiota in butyric acid metabolism.

In this study, we aimed to: (1) characterise gut microbiota and SCFA changes during and after colonoscopy; (2) identify bacteria encoding buk in human gut microbiota and investigate buk sequence variability among different bacteria; and (3) explore whether supplementation with butyric acid-producing *Clostridium butyricum* accelerates gut microbiome and SCFA stabilisation.

## Materials and methods

### Fecal samples and intestinal content collection

**Fig. 1:**
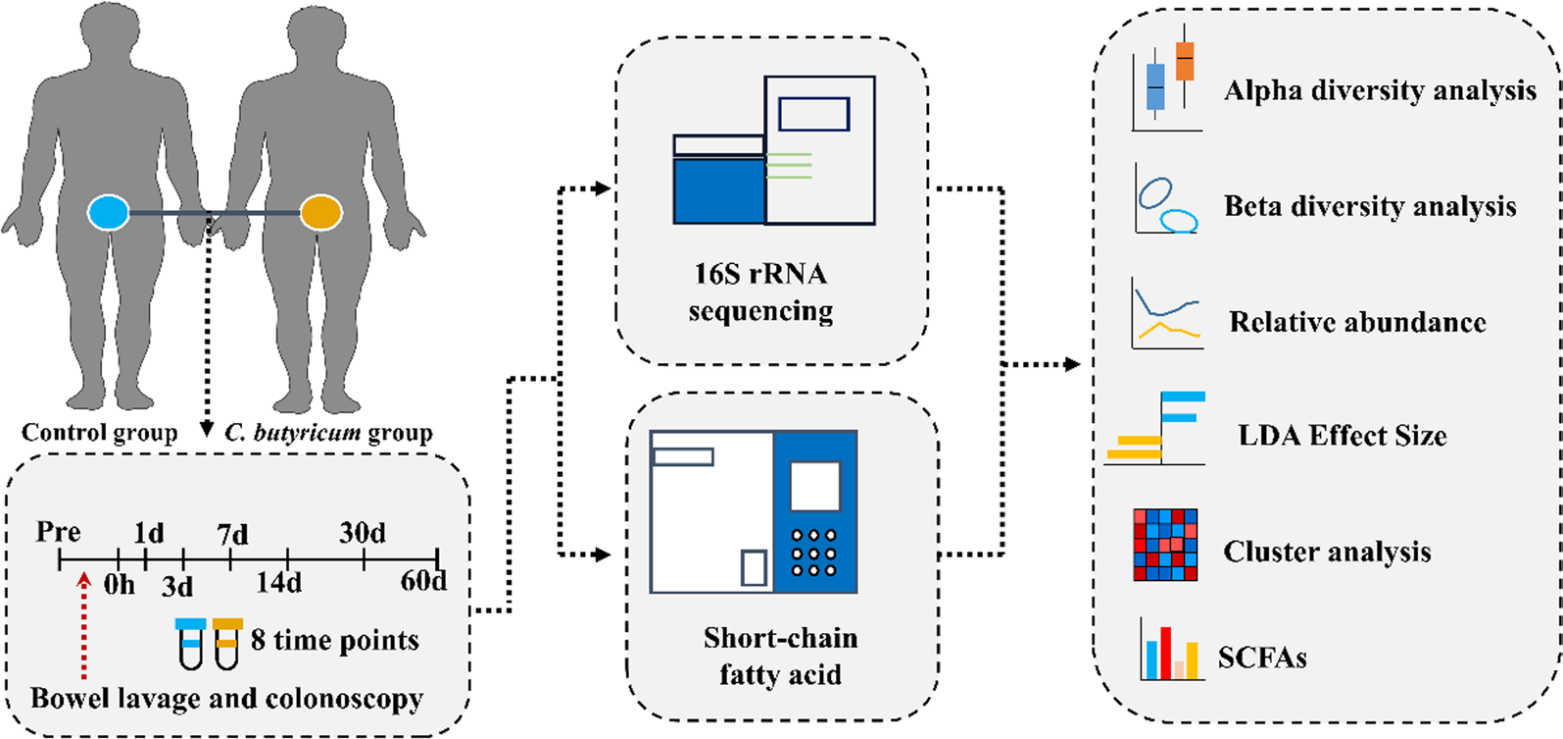
Overview of the study design and samples collection. *Pre* denotes the time point before bowel lavage, d 0 h denotes the time point during colonoscopy, while 1d, 3d, 7d, 14d, 30d, and 60d denote time points 1, 3, 7, 14, 30, and 60 days after colonoscopy

The research protocol was approved by the Ethics Committee of Nanfang Hospital, China (NFEC-2020-036). Eleven participants were enrolled in this study and their clinical characteristics are shown in Additional file 2: Table S1. The participants did not consume any food or medication containing probiotics within the 3 weeks prior to colonoscopy. In the group treated with *C. butyricum* (n = 7)*, C. butyricum* administration began immediately after colonoscopy and lasted for 20 days. Three pills of *C. butyricum* oral capsules, live (Eastsea Pharmacology, Qingdao, China) were administered to each participant twice a day. Biosamples were provided by the Department of Gastroenterology at Nanfang Hospital of Southern Medical University. Fecal samples were collected before bowel lavage (Pre), during colonoscopy (d 0 h) and after colonoscopy (day 1, 3, 7, 14, 30, and 60) with participants asked to bring a ‘fresh’ stool sample to the research location within 6 h after collection (Fig. 1, Additional file 1: Fig. S1 and Additional file 2: Table S1). Intestinal contents collected during colonoscopy were extracted using a sterile injection syringe. Twenty-five mL was centrifuged at 12,000 × g for 5 min at 4 °C. The supernatant was aspirated and discarded and the remaining pellet was stored at − 80 °C. Samples were temporarily stored at − 80 °C at the research location before transportation to LC-Bio Technology Co., Ltd. (Hangzhou, China).

### 16S ribosomal RNA (rRNA) gene sequencing

16S rRNA gene sequencing was performed as previously described [24]. Briefly, total genomic DNA from stool samples was extracted using a TIANamp Stool DNA Kit (TIANGEN Biotech, Beijing, China). The V4 region of the 16S rRNA gene was amplified using the forward primer 515F (5ʹ-GTGCCAGCMGCCGCGGTAA-3ʹ) and reverse primer 806R (5ʹ- GGACTACHVGGGTWTCTAAT-3ʹ). Eleven samples were sequenced on an Illumina NovoSeq6000 platform (Illumina, CA, USA) by LC-Bio Technology. The Quantitative Insights Into Microbial Ecology (QIIME2, v 2021.2) pipeline was employed to process the sequencing data, as previously described [25]. The lowest number of readings was normalized to the total number of reads obtained in the sequencing run. An operational taxonomic unit (OTU) table was further generated to record the abundance of each OTU in each sample and the taxonomy of these OTUs. OTUs containing less than 0.001% of total sequences across all samples were discarded. To minimize the difference of sequencing depth across samples, an averaged, rounded rarefied OTU table was generated by averaging 100 evenly resampled OTU subsets under 90% of the minimum sequencing depth for further analysis. Alpha diversity and principal coordinate analyses (PCoA) were conducted using a data visualisation web server ImageGP (www.ehbio.com/ImageGP/) [26].

### SCFA extraction and GC/MS analysis

Fecal samples and intestinal contents from eleven participants stored at − 80 °C were thawed at room temperature. One gram of sample was mixed with 1 mL OmniSolv (EMD Millipore Corporation, Billerica, MA, USA) pure water, homogenized for 10 min at 4 °C using a water bath sonicator (Sonics & Materials, Connecticut, USA), then centrifuged at 15,000 × g for 10 min at 4 °C. One mL of supernatant (fecal homogenate) was transferred into a new 4 mL centrifuge tube containing 10 µL of 50% (v/v) sulfuric acid to bring the pH of the fecal solution to 2. The sample was spiked with 10 µL 2-ethyl butyric acid (100 μg/mL in methanol) as an internal control. The acidified fecal homogenate was extracted by adding 2 mL methanol, vortexed for 30 s, incubated on ice for 5 min, then centrifuged at 10,000 × g for 10 min at 4 °C. The upper layer containing SCFAs was transferred to a new microtube containing anhydrous Na_2_SO_4_ to remove any residual water.

GC/MS analysis was performed using a 7890 B gas chromatograph/5977 single quadrupole (Agilent Technologies, Santa Clara, CA, USA) with a DB-FFAP125-3237 capillary column (30 m × 0.53 mm × 0.5 µm film thickness) (Agilent Technologies). The injector, ion source, quadrupole, and GC/MS interface temperatures (°C) were 200, 230, 200, and 280, respectively. The flow rate of helium carrier gas was maintained at 1 mL/min. One microliter of the derivatized sample was injected with a 3.5 min solvent delay and a split ratio of 10:1. The initial column temperature (90 °C) was held for 1 min, ramped to 200 °C at 12 °C/min and held for 2.33 min. Ionization was carried out in the electron impact mode at 70 eV. The MS data were acquired in full scan mode from m/z 40–400 with an acquisition frequency of 12.8 scans per second. The analytes were quantified in the selected ion-monitoring mode. The target ion (m/z) of the SCFAs was previously described [27]. Data were analyzed using Agilent MassHunter software and the SCFA content was calculated using external standard methods. The spiked recoveries were calculated using the following equation: recovery % = (final concentration − initial concentration)/spiked concentration × 100%.

### Taxonomic characterization of buk gene

The Culturable Genome Reference (CGR) database contains the reference genomes of 1,520 cultivated human gut bacteria, and it can be obtained from the NCBI database under accession code PRJNA482748 (https://www.ncbi.nlm.nih.gov/bioproject/PRJNA482748) [28]. De novo assembly was conducted according to a previous protocol. The genes and related proteins from these bacterial genomes were predicted using Prokka (v1.3) [29], and taxonomic information regarding these genes and proteins was directly extracted from the strain names. All *buk* sequences are shown in Additional file 3: Table S2. Pairwise amino acid sequence alignments and multiple alignments of *buk* sequences were performed using ClustalW in MEGA software (v11.0) [30]. Multiple sequence alignment was presented in Additional file 5. A phylogenetic tree was constructed using the maximum likelihood method and Kimura 2-parameter model in MEGA with 1000 bootstrap replications. An interactive tree of life (iTOL) (v5.0, https://itol.embl.de) was used to embellish the phylogenetic tree by adjusting the labels and colourisation [31]. The nine classified *buk* phylotypes represented were *buk-*T1-9. The n-*buk* represented bacteria with no *buk* gene in the genome.

### RNA isolation and RT-qPCR

Total RNA was extracted from bacteria (0.1 g) using TRIzol reagent. RNA was converted to cDNA using a reverse transcription kit. Gene expression was determined using qPCR SYBR Green Master Mix and the 7500 real-time quantitative PCR system (Applied Biosystems, Thermo Fisher Scientific, Waltham, MA, USA). 16S was used for normalisation. Relative quantification of the target genes was performed using the 2^−ΔΔCT^ method. The forward and reverse primers of *buk* were 5ʹ-TGCTGTWGTTGGWAGAGGYGGA-3ʹ and 5ʹ- GCAACIGCYTTTTTTGATTTAATGCATGG-3ʹ. The forward and reverse primers of 16S were 5ʹ-CCTACGGGNGGCWGCAG-3ʹ and 5ʹ-GACTACHVGGGTATCTAATCC-3ʹ.

### Statistical analysis

Statistical significance between changes in bacterial abundance at the phylum level was determined using Student’s t-test. Statistically significant differences between groups were determined using the Wilcoxon rank-sum test with false discovery rate correction. Cluster analysis was performed using Bray–Curtis similarity. Statistically significant differences in SCFAs and *buk* at different time points were calculated using the paired-samples t-test. Relationship analysis was performed using Pearson correlations. All analyses were performed using R version 3.6.1 and statistical significance was set at *p* < 0.05.

## Results

**Fig. 2:**
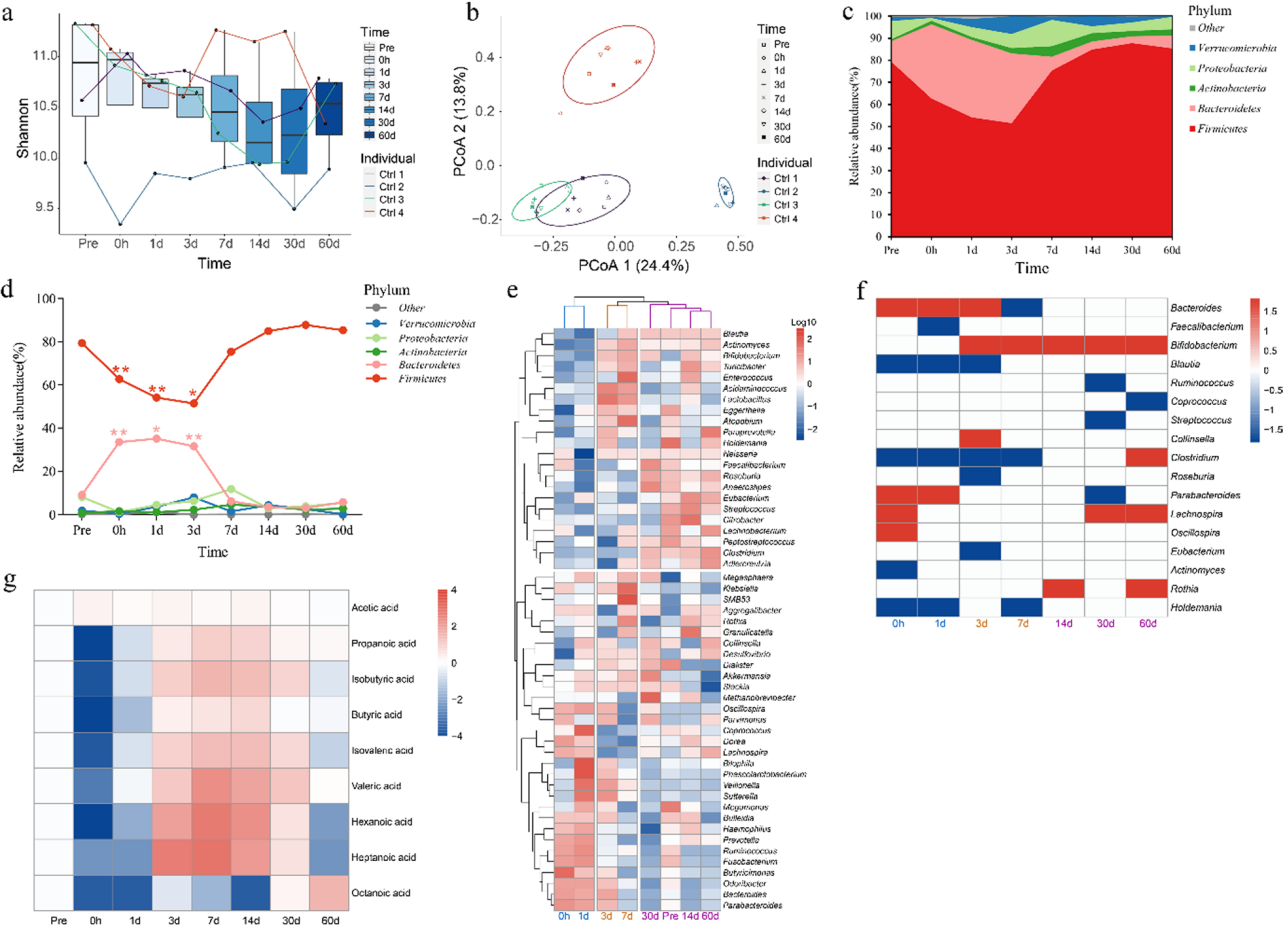
Longitudinal dynamics of human gut microbiota and SFCA in the control group. **a** Alpha diversity of control group gut microbiota over time. **b** PCoA analysis of control group gut microbiota at each time point. **c** Tracing the OTUs at each time point. Fecal samples of the former time point were taken as potential sources of the latter time point. The bands of each color indicate the different phylum. **d** The temporal changes of bacterial abundance at the phylum level and different time points. The significant points represent the changes from *Pre*. **e** Clustering of the gut microbiota at the genus level and different time points, a log10 transformation was performed on the OTU.** f** Significantly different genera at each time point, compared to the *Pre* time point with a *p*-value < 0.05 (using Wilcoxon rank-sum test), are shown. Colour in the heatmap represent z-values obtained from the Wilcoxon rank sum test. **g** The changes of SCFAs in fecal samples, normalized to *Pre*. *p < 0.05, **p < 0.01

Fecal samples and intestinal contents were collected from four healthy untreated subjects at nine time points. The microbial diversity in the fecal samples fluctuated significantly over time both during colonoscopy and after colonoscopy in all human subjects, and especially in Ctrl 2 (Fig. 2a). The microbiota of each subject clustered together at the different time points indicating conservation of the gut microbiota amongst individuals (Fig. 2b). The microbial community prior to colonoscopy served to determine species turnover (i.e., the change in microbial structure) after colonoscopy. *Firmicutes* appeared on day 3 after colonoscopy and *Bacteroidetes* increased during colonoscopy (Fig. 2c). *Firmicutes* and *Bacteroidetes* changed significantly during colonoscopy and on days 1 and 3 after colonoscopy (*P* < 0.05, Fig. 2d). Almost all bacterial phyla were at their highest or lowest abundances on day 3 and *Firmicutes* decreased by 30% (*p* < 0.05, Fig. 2d). The *Firmicutes* to *Bacteroidetes* ratio decreased during colonoscopy and on the third day after colonoscopy then returned to baseline levels on day 7 (Additional file 1: Fig. S2). There were three clusters based on the longitudinal gradient of gut microbiota: the early, middle, and mature phases with changes in genera change patterns within each phase (Fig. 2e). The bacteria that underwent changes in the early phase (0 h and 1 d) were bacteria with quick recovery; the bacteria that increased in the middle phase (3 d and 7 d) were primary bacteria and formed a foundation for the successive bacteria; and the bacteria that reproduced in the mature phases (14 d, 30 d and 60 d) but had not completely recovered at the end of this period were members of long-term changes (Fig. 2e). *Bacteria* requiring long-term recovery included *Bifidobacterium*, *Clostridium*, *Lachnospira*, and *Rothia* (*p* < 0.05, Fig. 2f).

Butyric acid, propanoic acid, hexanoic acid, and heptanoic acid levels decreased in stool samples during day one and recovered close to baseline levels 1 month after colonoscopy (Fig. 2g). Most SCFAs increased from 3 to 14 d after colonoscopy, however, acetic acid levels remained steady over the entire time period (Fig. 2g).

**Fig. 3:**
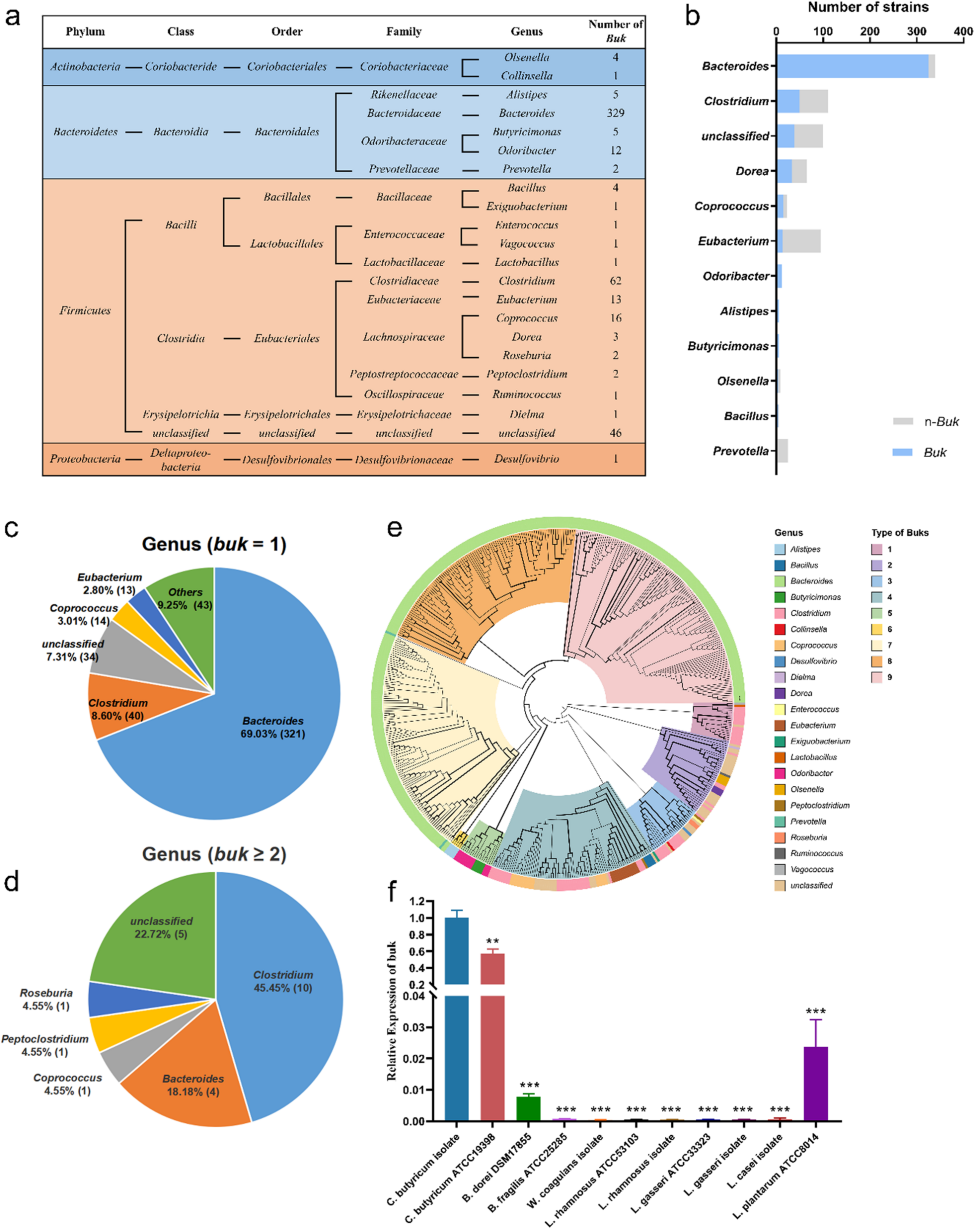
Taxonomic characterization of *buk*s obtained from the CGR database. **a** Taxonomic identification of *buk*s in different main genera. **b** Quantity of *buk*s containing bacterial strains at the genus level. **c** The proportion of bacterial strains at genus level with one *buk* gene. **d** The proportion of bacterial strains at genus level with two or three *buk* gene. **e** Taxonomic characterization of reclassified *buk*s. The width of the clade indicates the value of bootstrap, normalized to 0–1. **f** The mRNA level of *buk* in different bacterias. *p < 0.05, **p < 0.01, ***p < 0.001

Due to the long recovery time after colonoscopy, we hypothesised that *Clostridium* could be a good probiotic to help accelerate restoration of the gut microbiome and SCFAs. Bioinformatic analysis of *buk* gene was performed. Bacteria were assigned to 22 genera including *Bacteroides*, *Clostridium*, *Coprococcus*, *Eubacterium*, and *Odoribacter* from four phyla (Fig. 3a, Additional file 4: Table S3). More than half of the *buk* containing bacteria belong to the *Bacteroidetes* phyla (349 strains, 78.07%) and *Firmicutes* phyla (132 strains, 16.58%) (Additional file 1: Fig. S3). A total of 95.87 and 45.16% of the bacteria strains encoding *buk* belong to the *Bacteroides* and *Clostridium* genus, respectively (Fig. 3b). Meanwhile, 69.03% (321 strains) of *Bacteroides* and 8.60% (40 strains) of *Clostridium* strains encoded only one *buk* gene, while 45.45% (10 strains) of *Clostridium* and 18.18% (4 strains) of *Bacteroides* strains had two or three paralogous *buk* genes (Fig. 3c, d). This suggests that *Clostridium* strains have higher butyric acid production proficiency. The 515 *buk* genes in the CGR were classified into nine phylotypes by phylogenetic analysis with the number of sequences stated in parenthesis: *buk*—T1 (18), *buk*—T2 (37), *buk* -T3 (27), *buk* -T4 (79), *buk* -T5 (17), *buk* -T6 (5), *buk* -T7 (107), *buk* -T8 (104), and *buk* -T9 (120) (Fig. 3e). The results of qPCR indicated that two *C. butyricum* strains, not only the strain of *C. butyricum* used in this study, but also the strain of *C. butyricum* in the American Type Culture Collection, had significantly higher mRNA levels of *buk* compared with those in other probiotics (Fig. 3f).

**Fig. 4:**
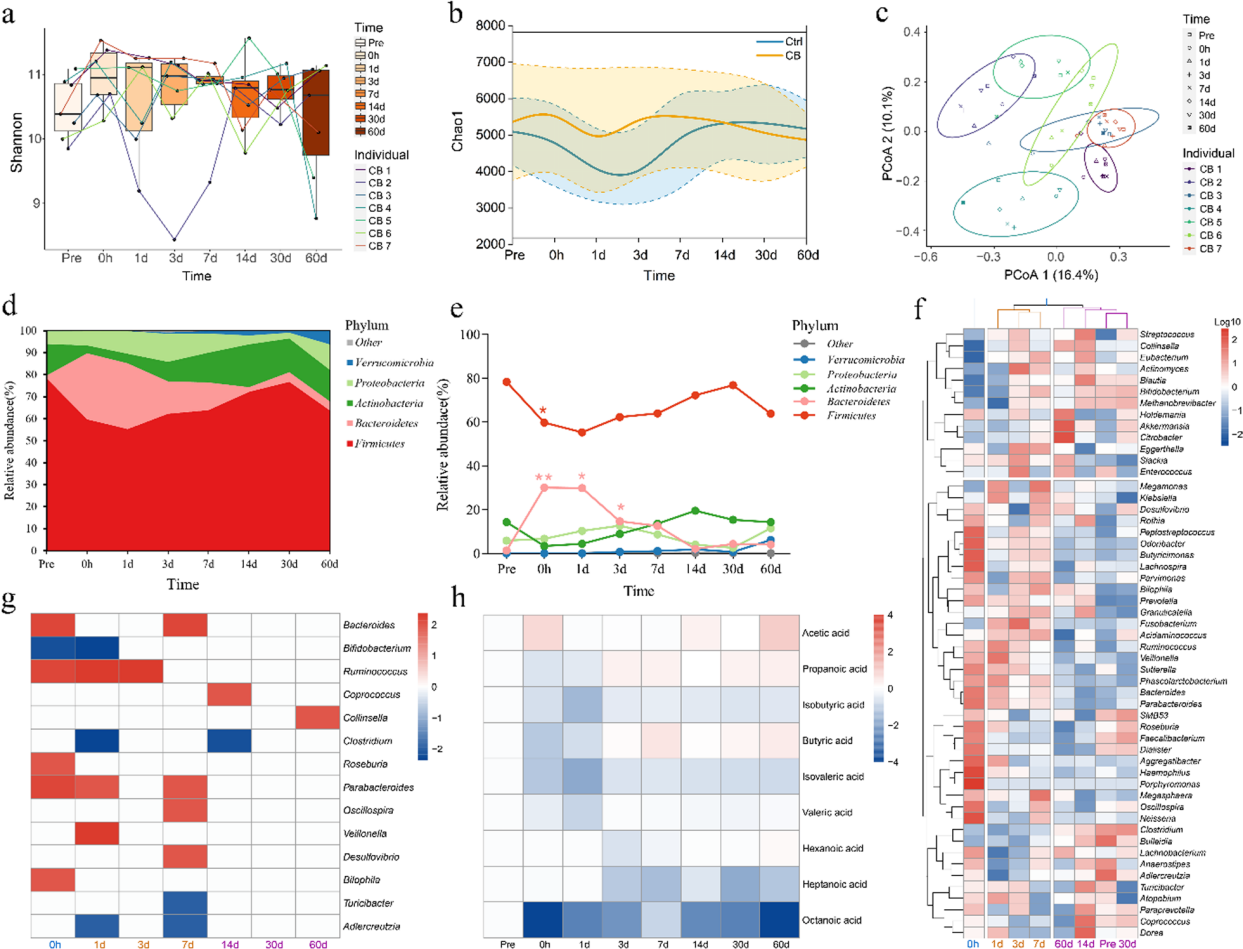
Longitudinal dynamics of human gut microbiota and SFCA in the *Clostridium Butyricum* (CB) treated group. **a** The alpha diversity of the CB group gut microbiota over time. **b** Chaos diversity index of gut microbiota in the control group and the CB group. **c** The PCoA analysis of CB group gut microbiota at each time point. **d** Tracing the OTUs in each time point. The bands of each color indicate the different phyla. **e** The temporal changes of bacterial abundance at the phylum level. The significant points represent changes from *Pre*. **f** Clustering of the gut microbiota at the genus level and different time points, a log10 transformation was performed on the OTU. **g** Significantly different genera at each time point, compared to the *Pre* time point with a p-value < 0.05 (using Wilcoxon rank-sum test), are shown. Colours in the heatmap represent z-values obtained from the Wilcoxon rank sum test. **h** The changes of SCFAs in fecal samples, normalized to *Pre.* *p < 0.05, **p < 0.01

*C. butyricum* is the only *Clostridium* species that has been commercialized and approved by the National Medical Products Administration in China. The Fecal microbial alpha diversity slightly fluctuated during and after colonoscopy in human subjects compared with the former Control group, except for CB 2 (Fig. 4a and b). The microbiota of each subject clustered together at different time points (Fig. 4c). *Firmicutes* decreased by 16.1% in *C. butyricum-*treated subjects on three days after colonoscopy compared with 27.9% in the control, while *Bacteroidetes* increased by 13.4% on three days after colonoscopy compared with 22.4% in control subjects (Fig. 4d and e). *Firmicutes* and *Bacteroidetes* changed significantly from during and 1d after colonoscopy (*P* < 0.05, Fig. 4e). Almost all of the bacterial phyla were at their highest or lowest abundance after 1 d in the *C. butyricum-*treated subjects, which is 2 d ahead of the Control group (Fig. 4e). The ratio of *Firmicutes* to *Bacteroidetes* decreased during the first day after colonoscopy and returned to baseline levels on the third day (Additional file 1: Fig. S4). Three clusters were grouped based on the longitudinal gradient of fecal samples: early phase (0 h), middle (1 d, 3 d and 7 d) phase and mature (14 d, 30 d and 60 d) phase (Fig. 4f). The middle gut microbiota phase indicated a faster recovery of gut microbiota changes beginning from 1 d. *Bacteroides*, *Ruminococcus*, *Parabacteroides*, *Oscillospira*, *Veillonella*, and *Desulfovibrio* increased in the middle phase as the first colonizers, while *Coprococcus* and *Collinsella* increased in the mature phase as successive colonizers (*p* < 0.05, Fig. 4g). Butyric acid and other SCFAs decreased during colonoscopy and recovered to baseline levels after 1–3 d, which is four days faster than the Control group (Fig. 4h). Collectively, these results show that *C. butyricum* accelerates the restoration of gut microbiota balance and provides SCFAs in the gut.

**Fig. 5:**
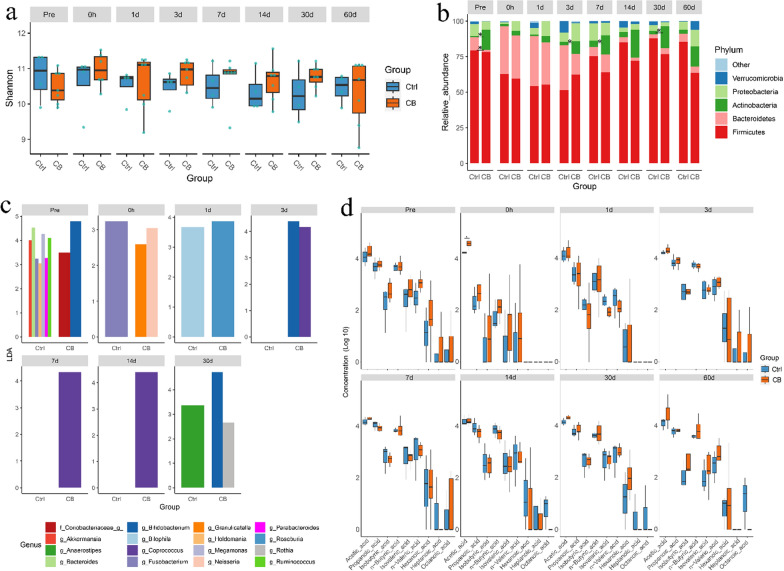
Comparison of human gut microbiota and SCFAs in the control (Ctrl) and *C. Butyricum* (CB) treated groups. **a** The Shannon diversity index of the two groups over time. **b** Changes in bacterial abundance at the phylum level. Significant points indicate a statistically significant difference in phylum levels between the two groups at each time point. **c** Genera that differed significantly between the two groups at each time point were identified using LefSe analysis, with only the genera with LDA > 2 and *p* < 0.05 shown. **d** Changes in SCFA levels in fecal samples at different time points. *p < 0.05

Furthermore, a direct statistical analysis was conducted between the control group of four participants and the experimental group of seven individuals supplemented with *C. butyricum*. The microbial diversity between the control and *C. butyricum* groups was not significantly different, as shown in Fig. 5a. However, the abundance of the phylum Actinobacteria was significantly higher, while the abundance of Bacteroidetes was significantly lower in the *C. butyricum* group at time point Pre (Fig. 5b). Notably, the abundance of Actinobacteria remained significantly higher in the *C. butyricum* group at 3 d, 7 d, and 30 d after colonoscopy when compared to the control group (Fig. 5b). Using LEfSe analysis, the *Coriobacteriaceae* family and *Bifidobacterium* genus were significantly higher in the *C. butyricum* group at time point Pre, while the *Granulicatella* and *Neisseria* genera were significantly higher during colonoscopy. The *Roseburia* genus was significantly higher in the *C. butyricum* group at 1 d, and the *Bifidobacterium* and *Coprococcus* genera were significantly higher at 3 d, with the *Coprococcus* genus continuing to maintain higher levels at 7 d and 14 d. At 30 d, *Bifidobacterium* and *Rothia* genera were significantly higher in the *C. butyricum* group (Fig. 5c). No statistically significant differences were observed in bacteria obtained from the LEfSe analysis at day 60d. Furthermore, the SCFA concentrations of the two groups were compared. The acetic acid levels in the *C. butyricum* group were significantly higher than those in the control group at d 0 h (Fig. 5d). Although there was no statistically significant, acetic acid levels in the *C. butyricum* group were higher than those in the control group at 30 d and 60 d. Hexanoic acid levels in the *C. butyricum* group were higher than those in the control group at 30 d, and valeric acid levels in the *C. butyricum* group were higher than those in the control group at 60 d (Fig. 5d).

**Fig. 6:**
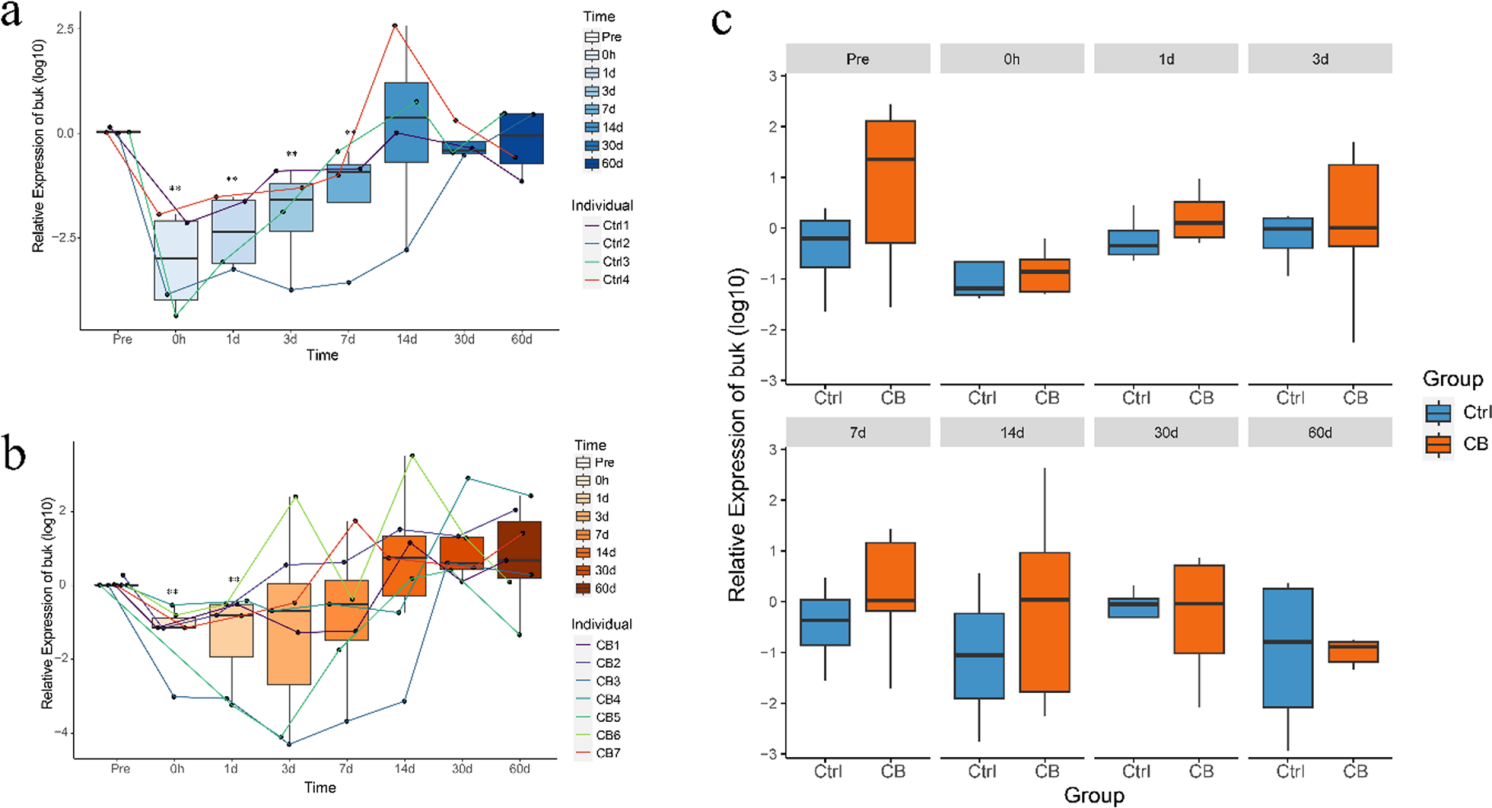
Transcription levels of *buk* in eleven subjects across all time points. **a** Transcription levels of *buk* in the control group at all time points, with all data compared to time point *Pre* and performed log10 transformation. Paired sample t-test was performed with *Pre* and other time points. **b** Transcription levels of *buk* in the *C. Butyricum* group at all time points, with all data compared to the *Pre* performed log10 transformation. Paired sample t-test was performed with *Pre* and other time points. **c** Transcription levels of *buk* in two groups at various time points. All data performed log10 transformation

The fecal mRNA levels of the *buk* gene were examined in all study participants. The qPCR results revealed a noticeable reduction in the *buk* mRNA levels immediately following colonoscopy, followed by a gradual recovery over time (Fig. 6a, b). In subjects who did not receive *C. butyricum* supplementation, the *buk* gene levels returned to their baseline levels at fourteen days after the procedure (Fig. 6a). Conversely, in the *C. butyricum*-treated group, the *buk* gene levels reached the baseline as early as three days after the colonoscopy and steadily increased with continued consumption of the supplement (Fig. 6b). Additionally, a comparison of *buk* abundance between the two groups at *Pre*, 0 h,1 d, 7 d, 14 d revealed a higher level of *buk* in the *C. butyricum* group compared to that in the control group, although there is no statistical difference (Fig. 6c).

## Discussion

There are few studies that have analyzed *buk* paralogs in bacterial genomes. We systematically analyzed 1520 cultivated bacteria in the human gut and identified that *Bacteroides* and *Clostridium* species have the *buk* gene indicating that they have the potential to generate butyric acid which is consistent with previous studies using these cultures [14–16].

SCFAs play an essential role in intestinal mucosal repair [11, 12]. However, little is known about SCFA changes during or after colonoscopy. Butyric acid is the most common SCFA generated by gut microbiota. In this study, dynamic changes in SCFAs including butyric acid were observed one day after colonoscopy, and SCFAs may require 1 week to recover to baseline levels. Therefore, complications arising one week after colonoscopy may be alleviated by supplying SCFAs, fatty acid salts, or other biological agents to patients.

Despite several studies showing changes in intestinal microbiota two-eight weeks after colonoscopy in adults [32], little attention is paid during the early stages (1–7 d) after colonoscopy. Bowel preparation before colonoscopy resulted in substantial immediate changes in the intestinal microbiota which persisted 2 weeks and 4 weeks after colonoscopy in a randomized controlled trial involving 23 healthy subjects [33]. The total microbial load significantly decreased and nearly one-fifth of participants lost subject-specificity of their gut microbiota. There was a significant decrease and increase in *Firmicutes* and *Proteobacteria* abundance, respectively immediately after colon cleansing and 1 month after colonoscopy using 16S rRNA Ion Torrent profiling of fecal samples from 10 patients [34]. In this study, dynamic changes in gut microbiota were observed between the first and seventh days after colonoscopy using 16S rRNA sequencing. There was a significant decrease in *Firmicutes* and an increase in *Bacteroidetes* after 3 days. The middle phase (3 d) was distinct from the early (1 d) and mature (7 d) phase. *Bifidobacterium*, *Clostridium*, *Lachnospira*, and *Rothia* were identified as bacterial members involved in long-term changes. The use of these bacteria as a probiotic was hypothesized to accelerate the restoration of the gut microbiota balance.

Bowel preparation before colonoscopy causes microbiota-related symptoms for days, resulting in the loss of working days. Therefore, there is a need to rapidly counterbalance microbiota alterations. Probiotics may offer a potential therapeutic option to restore the altered gut microbiota. For example, tablets containing *Bifidobacterium tetragenous* restore intestinal dysbacteriosis on the seventh day after bowel preparation [35]. A combination of *Lactobacillus acidophilus* and *Bifidobacterium* species producing lactic acid results in significant pain reduction following colonoscopy, shortened from 1.99 to 2.78 days [36]. However, a probiotic mixture (*Lactobacillus plantarum*, *Lactobacillus lactis subspecies cremoris*, and *Lactobacillus delbrueckii*) only has a benefit in patients with pre-existing symptoms and does not help ease post-procedural symptoms in healthy subjects [37]. In this study, patients supplemented with *C. butyricum* had significantly improved microbiotal balance with butyric acid levels restored significantly faster than those in untreated patients and *B. tetragenous* treated patients as previously reported (see Additional file 5).

This study has three main limitations. First, only 11 subjects provided continuous fecal samples which may have resulted in sample bias, and additional subjects are required to draw more accurate conclusions. Conducting these types of studies requiring consistent fresh fecal samples was challenging given that individuals with normal colonoscopy results do not visit hospitals frequently. We proposed recruiting college students or clinical staff members as potential study participants to increase the sample size. Second, the symptoms of the participants after colonoscopy were not recorded. Third, the molecular mechanism by which *C. butyricum* resulted in quicker recovery and stabilisation of gut microbiota and fecal SCFAs remains unknown. This unresolved issue will be the focus of future research.

This study revealed the changes in microbiota and SCFA metabolites from stool samples in the early stages (one week) after colonoscopy. *C. butyricum* produces more butyric acid and other SCFAs than other probiotics and has a potential therapeutic role in restoring the balance of the gut microbiome. Routine application of *C. butyricum* after colonoscopy might be a potentially effective method to limit the complications associated with this procedure. Future investigations should focus on improving restoration of the gut microbiome by supplying probiotics and prebiotics.

### Supplementary Information

**Additional file 1: Figure S1.** Faecal samples and intestinal contents were collected from 11 subjects at 8 time points before, during and 60 days after colonoscopy. NA denotes an incurred sample loss. **Figure S2.** The ratio of Firmicutes and Bacteroidetes showed the longitudinal fluctuation patterns of gut microbiota in the Control group. **p < 0.01. **Figure S3.** Quantity of buks containing bacteria stains at the phylum level. **Figure S4.** The ratio of Firmicutes and Bacteroidetes showed the longitudinal fluctuation patterns of gut microbiota in the Clostridium Butyricum group. **p < 0.01.

**Additional file 2: Table S1.** Clinical characteristics of 11 subjects.

**Additional file 3: Table S2.** Sequences of buk in 1520 bacterias.

**Additional file 4: Table S3.** Taxonomic identification of buks in CGR database.

**Additional file 5.** Multiple sequence alignment.

## Data Availability

Raw data of 16S rRNA sequencing in this study are available in the NCBI database under accession code PRJNA827030. (https://www.ncbi.nlm.nih.gov/bioproject/PRJNA827030).
