# Supplementary material for: Gut microbiota dynamics and fecal SCFAs after colonoscopy: accelerating microbiome stabilization by Clostridium butyricum
Source: J Transl Med. 2024 Mar 1;22:222. doi: 10.1186/s12967-024-05031-y (PMC10908214; doi:10.1186/s12967-024-05031-y)
Supplement: Supplementary file 1 — Additional file 1: Figure S1. Faecal samples and intestinal contents were collected from 11 subjects at 8 time points before, during and 60 days after colonoscopy. NA denotes an incurred sample loss. Figure S2. The ratio of Firmicutes and Bacteroidetes showed the longitudinal fluctuation patterns of gut microbiota in the Control group. **p < 0.01. Figure S3. Quantity of buks containing bacteria stains at the phylum level. Figure S4. The ratio of Firmicutes and Bacteroidetes showed the longitudinal fluctuation patterns of gut microbiota in the Clostridium Butyricum group. **p < 0.01. [file 12967_2024_5031_MOESM1_ESM.docx]

**
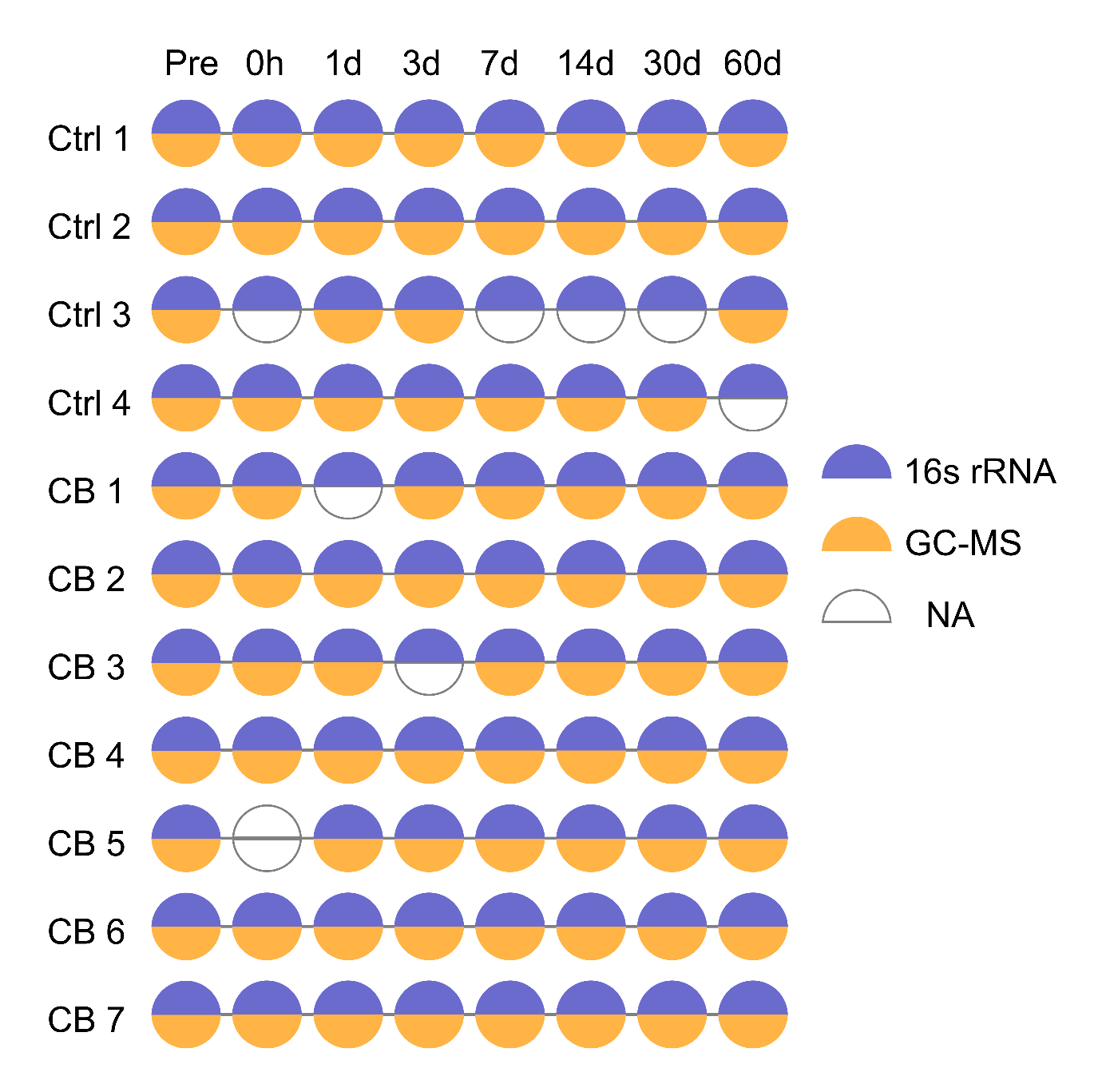
**

**Supplementary figure 1.** **Faecal samples and** **intestinal contents were collected from 11 subjects at 8 time points before, during and 60 days after colonoscopy.** NA denotes an incurred sample loss.


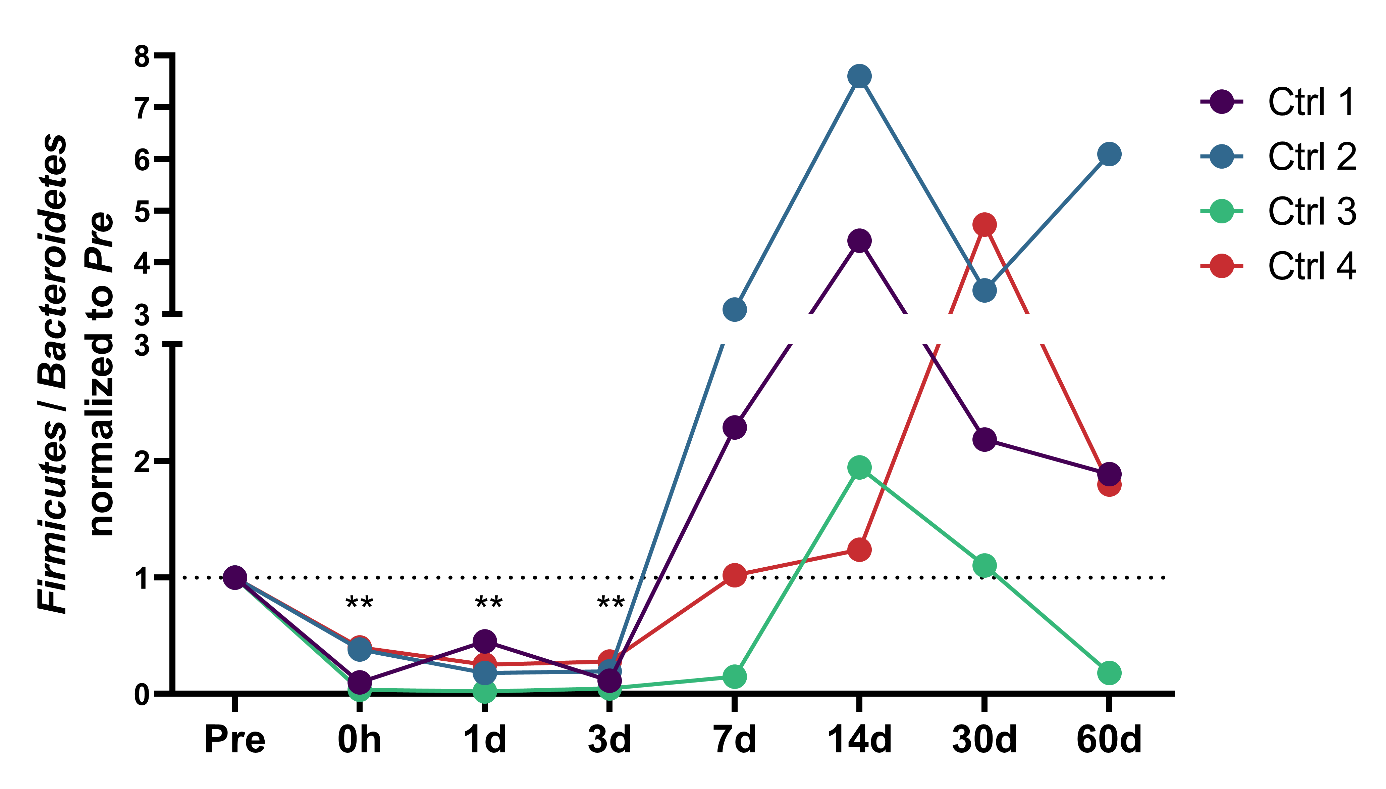


**Supplementary figure 2. The ratio of *Firmicutes* and *Bacteroidetes* showed the longitudinal fluctuation patterns of gut microbiota in the Control group. **p < 0.01.**

**
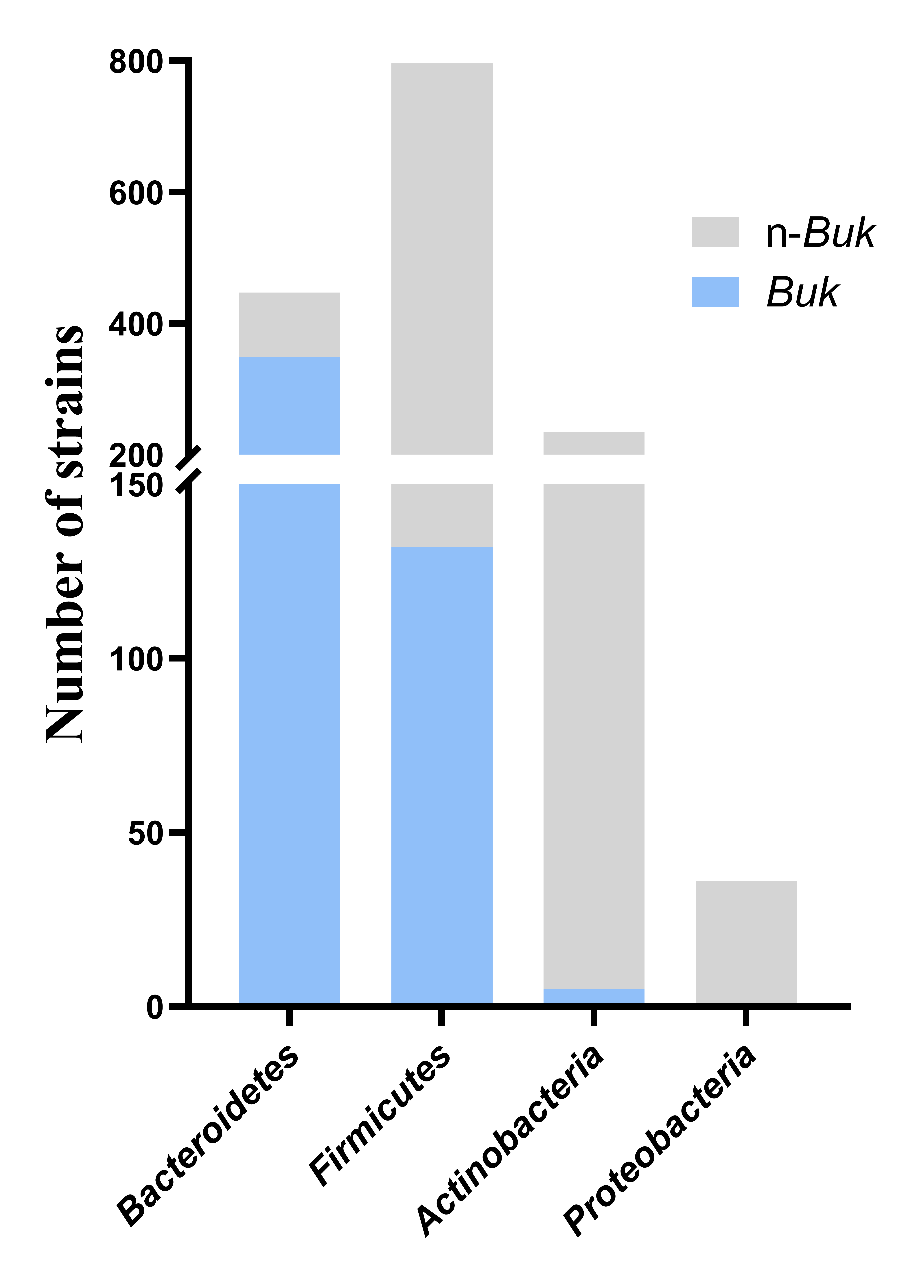
**

**Supplementary figure 3. Quantity of buks containing bacteria stains at the phylum level.**


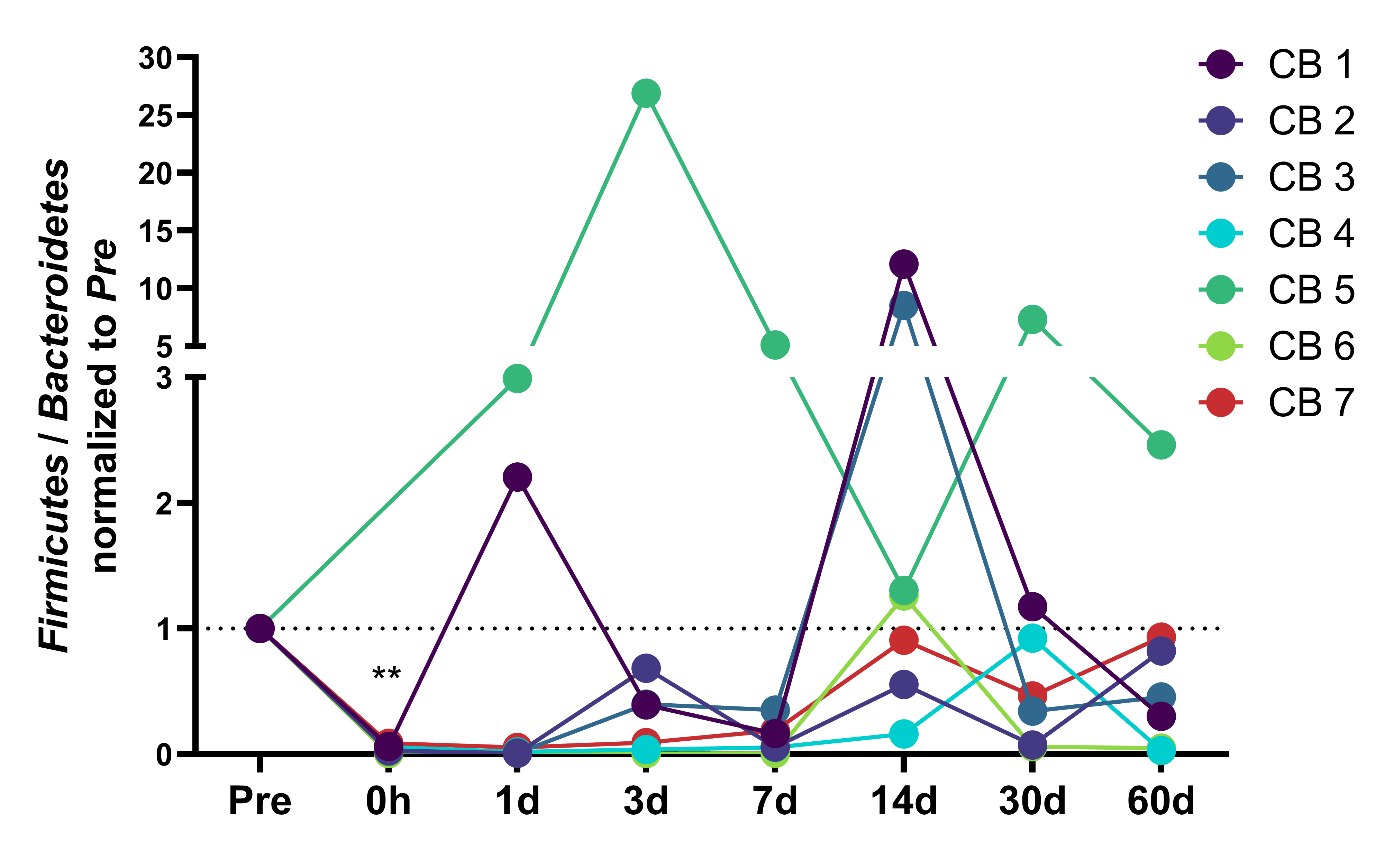


**Supplementary figure 4. The ratio of *Firmicutes* and *Bacteroidetes* showed the longitudinal fluctuation patterns of gut microbiota in the *Clostridium Butyricum* group. **p < 0.01.**
